# Evaluation of synergistic approach of spinel cadmium–copper nanoferrites as magnetic catalysts for promoting wastewater decontamination: Impact of Ag ions doping

**DOI:** 10.1007/s11356-023-27170-3

**Published:** 2023-05-05

**Authors:** Ahmed H. Mangood, Ali H. Gemeay, Mohamed M. Abdel-Galeil, Eman Sh. Salama, Reda E. El-Shater

**Affiliations:** 1 Chemistry Department, Faculty of Science, Menofia University, Shabien Elkom, Egypt; 2 Chemistry Department, Faculty of Science, Tanta University, Tanta, Egypt; 3 Physics Department, Faculty of Science, Tanta University, Tanta, Egypt

**Keywords:** Spinel ferrites, Synergistic effects, Ag^+^ doping, Advanced oxidation process, Wastewater treatment

## Abstract

Metal substitution is an efficient strategy to improve the catalytic activity of ferrite-based catalysts. In this study, Cd_0.5_Cu_0.5−*x*_Ag_*x*_Fe_2_O_4_ (where 0 ≤ *x* ≤ 0.5) ferrites were fabricated via a simple co-precipitation method. The influence of the silver ions on the structural, magnetic, and catalytic characteristics of the spinel nanoparticles, as well as on their morphology, was examined. X-ray diffractograms revealed a crystalline cubic spinel structure with crystallite sizes in the nanoregime (7–15 nm). The saturation magnetization reduced from 29.8 to 2.80 emu as the Ag^+^ doping increased. Two prominent absorption bands were visible in Fourier-transform infrared spectra at 600 cm^−1^ and 400 cm^−1^, respectively, and they belonged to the tetrahedral (A) and octahedral (B) sites. The samples were then used as catalysts for the oxidative breakdown of the typical organic contaminant indigo carmine dye (IC). The catalytic process followed the first-order kinetic model, and the rate constant increased from 0.007 to 0.023 min^−1^ with increasing of Ag^+^ doping. Cd_0.5_Cu_0.5-*x*_Ag_*x*_Fe_2_O_4_ exhibited excellent catalytic performance in the pH range of 2–11, which means that they are promising efficient and stable materials for Fenton-based alkaline wastewater treatment. Finally, the pathway includes, HO^•^, HO_2_^−•^, and O_2_^−^• as oxidants resulted from the synergistic effects of Fe^3+^, Cu^2+^, and Ag^+^, with H_2_O_2_ and surface hydroxyl groups have been proposed.

**Graphical Abstract:**

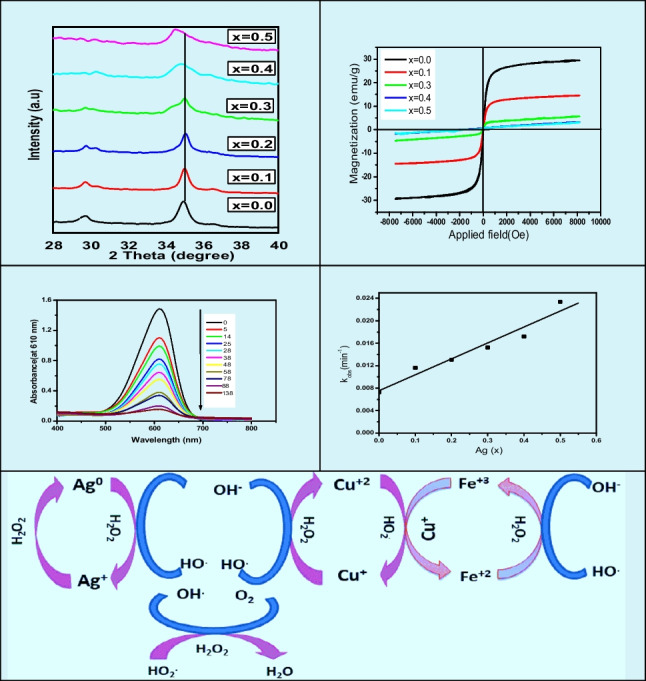

## Introduction

Many industrial catalysts are based on noble metals or metal oxides. At relatively low temperatures, the noble metal-based ones are highly effective but also vulnerable to sintering and susceptible to poisoning. Access to resources for safe, dependable, and uncontaminated drinking water is humanity’s essential requirement for a thriving and stable society. Environmental contamination has increased as a result of the extending of agricultural and industrial operations and the careless use of resources (Hosseini et al. 2019). Because of the massive amounts used every day, water pollution caused by organic dyes has been a source of concern in recent years resulting in environmental and health issues (Gonçalves et al. 2020). Indigo carmine is a common anionic dye from the indigoid family used in cosmetics, medicines, and food manufacturers (Dayana et al. 2021). It could be released from a variety of sectors, including textile, paper, and plastics processors, causing major pollution in aquatic bodies. It was resistant to oxidation and cracking because of its symmetrical structure and stable characteristics (Bouteraa et al. 2020).

Several studies have recently focused on the removal of dye-based hazardous and carcinogenic contaminants from industrial effluent. Organic dye pollution in wastewater has been reduced using a variety of techniques based on physical–chemical characteristics (Gonçalves et al. 2020). Various physical, chemical, and biological techniques have been widely used to treat these contaminations from wastewater (Shamsi Kasmaei et al. 2020). The adsorption process has been reported to exhibit high color removal efficiency and adsorbent regeneration capacity, but its application is impeded by sludge production (Mcyotto et al. 2021). The development of more effective and promising methods for degrading contaminants in industrial wastewater, known as advanced oxidation processes (AOPs), has recently attracted significant scientific attention. Chemical wastewater treatment processes that use advanced oxidation techniques can completely mineralize organic pollutants into CO_2_, water, and inorganic compounds, or at the very least, transform them into more benign chemical species.

The H_2_O_2_-based AOP has significant development potential because H_2_O_2_ is very easy to obtain and is inexpensive (Wu and East 2022). Advanced oxidation techniques involve the generation of enough hydroxyl radicals to oxidize organic contaminants (Alalm 2015; Girón-Navarro et al. 2021; Su et al. 2021). Superoxide and hydroxyl or SO_4_^•–^ radicals are unstable and highly reactive in most situations; appear to be the main oxidant species, even though other species may also contribute to degradation (Mehta et al. 2021; de Souza et al. 2021).

The design of multimetal ion catalyst featuring robust synergistic interactions provides new insight into the remediation of organic pollutants in wastewater. Metal ferrites are ceramic substances made out of iron oxide in the form of MFe_2_O_4_, where M stands for divalent transition metallic ions (Jaid et al. 2020). Due to the low cost, stable structure, and advantageous magnetic, electrical, and mechanical properties of nano-metal ferrites (Rajeevgandhi and Sivagurunathan 2019;Ansari et al. 2018; Kafshgari et al. 2019), they have been used as catalysts (Akhtar et al. 2016; Kulkarni and Mathad 2021; Vijayaraghavan et al. 2016). The use of ferrite nanoparticles as magnetic catalysts is very beneficial. Thus, we aimed to improve their catalytic efficiency based on the types of metal constituents, the variation of their ratios, and the simplicity of the synthesis method which are the crucial points of research. Furthermore, its magnetic nature makes them magnetically separable from the reaction mixture in a convenient manner.

Many factors affected ferrite characteristics including preparation conditions, size, microstructure, heat treatment, synthesis method, and cation distribution (Kulkarni and Mathad 2021; Rao et al. 2020). Ferrites have been fabricated with several methods (Amin et al. 2021; Rao et al. 2020; Yanjiao et al. 2021; Kulkarni et al. 2019; Shahid et al. 2017; Ateia et al. 2020; Patila et al. 2015). The co-precipitation method is one of the best synthesis procedures due to its many benefits, including high homogeneity and small particle size (Nabi et al. 2021). This method may also create ultrafine, high-purity, crystalline, and high-yield nanoparticles (Abdolmohammad-zadeh and Ayazi 2022).

Normal spinel ferrite CdFe_2_O_4_ is technically important and suitable for electrical switches (Nagarajan and Thayumanavan 2018; Sagadevan et al. 2017; Anjum et al. 2020; Mahmoud and Abd-Elrahman 2012; Yu et al. 2018). Cu^2+^ and Ag^+^ ions were often selected among various dopants (Mahmoud and Abd-Elrahman 2012; Jauhar et al. 2014). CuFe_2_O_4_ showed the best catalytic oxidation toward methylene blue with 90.5% within 3 h and rate constant reaching 0.794 h^−1^ (Dang et al. 2016). In addition, 99% of chlortetracycline was removed at a dosage = 2.0 g L^−1^ of Cu/Fe_3_O_4_ within 90 min (Liu et al. 2020). The catalytic combustion of acetone, ethanol, and methanol was achieved in the presence of Cu- and Ni-ferrite powders as catalysts. Multiple valences of Cu and Ni ions were suggested as a likely reason (Rezlescu et al. 2013).

Many silver compounds have been widely used in a variety of reactions, including isomerization, oxidation, catalytic, biological, optical, and photocatalytic reactions (Hosseini et al. 2016). Silver-doped ferrite thin films have been investigated and discovered that as the Ag + content increased, the saturation magnetization dropped (Zeehan et al. 2019). Silver-doped ferrite compounds such as silver-doped cobalt ferrite (Mahajan et al. 2019), silver magnetite nanocomposite (Jalali et al. 2017), Ag–Cu ferrite nanoparticles (Gomes et al. 2018), and silver ferrite–graphene nanocomposites (Hosseini et al. 2016) exhibited antibacterial activity against gram-positive and gram-negative strains. Moreover, silver nanoparticles (AgNPs) were employed as catalyst for the degradation of RhB and MO dyes in the presence of NaBH_4_ as reducing agent (Vankdoth et al. 2022). In addition, Ag–M_1-*x*_Fe_2+*x*_O_4_ (M = Co, Ni, Mn, Zn) nanocomposites display high activity in the epoxidation of styrene using tert-butyl-hydroperoxide (TBHP) as the oxidant (Zhang et al. 2009). The catalytic performance of 4-nitrophenol degradation via CuO has been improved after doping by Ag, decreasing the time of the total degradation from 60 to 25 min (Menazea and Mostafa 2020).

In the present study, Cd_0.5_Cu_0.5-*x*_Ag_*x*_Fe_2_O_4_ spinel ferrite nanoparticles were synthesized for the first time via co-precipitation method. The crystalline structure, morphology, and magnetization behavior were examined. Then, their catalytic performance for IC degradation using H_2_O_2_ as an environmentally benign oxidant was investigated. Additionally, the effects of several operational parameters were investigated, including pH, temperature, dye concentration, and catalyst dosage. The results could contribute to a better understanding of the synergistic role played by Ag^+^ ion doping and the different divalent ions in the catalytic activity of ferrite nanoparticles, which is very important in their use in wastewater treatment applications.

## Materials and methods

### Materials

Without further purification, all of the chemicals were of analytical quality. Cd (NO_3_)_2_.4H_2_O (98%), Fe(NO_3_)_3._9H_2_O (98%), Cu(NO_3_)_2._6H_2_O (98%), AgNO_3_ (99%), and IC were supplied from Lobe (India). NaOH and NH_4_OH were provided from ADWIC (Egypt). H_2_O_2_ was obtained from Merck (Germany).

### Sample preparation

The Cd_0.5_Cu_0.5-*x*_Ag_*x*_Fe_2_O_4_ spinel nanoferrites were prepared via coprecipitation. Cd(NO_3_)_2_ (0.5 mol L^−1^), AgNO_3_ (*x* mol L^−1^), Cu(NO_3_)_2_ (0.5–*x* mol L^−1^), and Fe(NO_3_)_3_ (0.6 mol L^−1^) were dissolved in distilled water and mixed together in a beaker with (1:2) molar ratio. Then NaOH (3 mol L^−1^) was then added drop by drop until a precipitate was formed. After being heated for 2 h at 80 °C, the reaction mixture was constantly agitated for 1 h at room temperature. After drying, the samples were then magnetic decanted and repeatedly washed with distilled water to create soft ferrite particles. The powders were dried overnight at 80 °C. Afterward, the synthesized samples were characterized with various analytical techniques.

### Sample characterization

Powder X-ray diffractometry (XRD) was used to examine the crystalline structure of the prepared samples (by using an Ultimate IV diffractometer (Rigaku, Japan)) operated at 40 kV and 20 mA over a 2*θ* range of 10–70° and using CuKα radiation (*λ* = 1.5406 Å). Using a field emission scanning electron microscopy (FESEM) instrument, the surface morphology was examined (SU8000 Type II, Hitachi). A transmission electron microscope (TEM) instrument (JEM-2100F, JEOL, Japan) operating at 200 kV was used to analyze the nanoparticle morphology. A high-performance double beam spectrophotometer (T80 +) with an electronic temperature controller was used to record ultraviolet–visible (UV–Vis) spectrum data. A thermogravimetric analysis (TGA) was carried out with a Rigaku ThermoPlus EVO2 system from room temperature to 800 °C at a heating rate of 10 °C in an airflow atmosphere. Magnetic properties were measured by using VSM DMS-880 hand-made, Physics Department, Faculty of Science, Tanta University, Egypt.

### Catalytic test

The catalytic reaction of IC was performed adding 10 mg of the synthesized ferrite catalyst to a solution of IC (10^−4^ mol L^−1^) and H_2_O_2_ (0.079 mol L^−1^); then the mixture was continuously stirred in a shaker water thermostat at a speed of 120 rpm. The reaction was monitored by taking samples at regular time intervals and the absorbance of IC at *λ*_max_ = 610 nm at any time *t*, and was traced by the UV–Vis spectrophotometer, which was decreased with time and indicates a decrement in the IC concentration. The IC consumption with time was determined by Eq. (1):

1 $$\mathrm{\%\; IC\; consumption}=\frac{{(A}_{o}-{A}_{t})}{{A}_{o}}x 100$$

where *A*_o_ and *A*_*t*_ represent the absorbance at time = 0 and any time *t*, respectively. These results confirm that the catalytic reaction follows the pseudo-first-order kinetic model with regard to the IC concentration, and its rate constant (*k*) was calculated by Eq. (2).

2 $$\left(\mathrm{k}\right)=\frac{1}{\mathrm{t}}\mathrm{Ln }\frac{{c}_{0} }{{c}_{t}}$$

## Results and discussion

### Characterization

#### XRD results

**Fig. 1:**
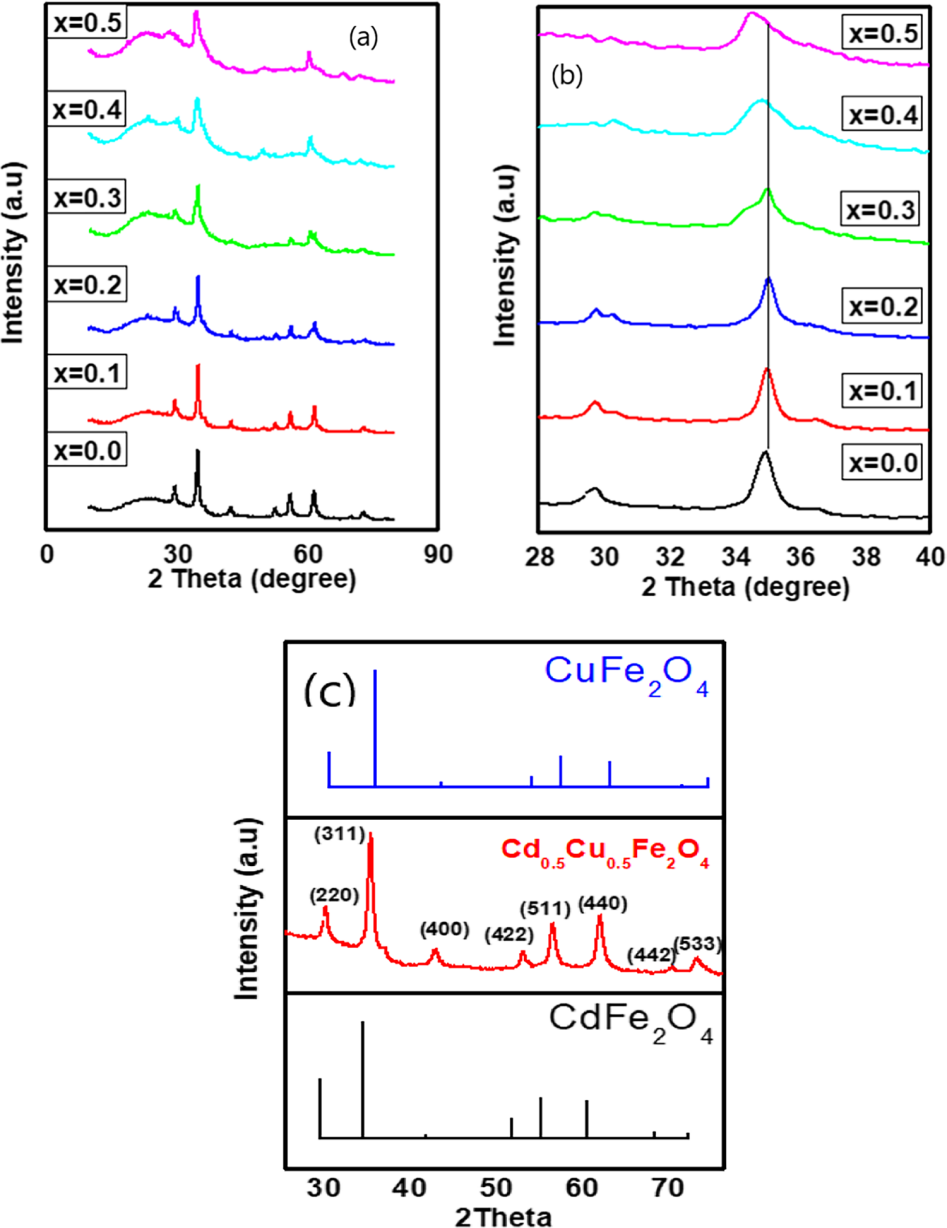
X-RD patterns of ferrites samples (**a**), focusing on (311) peak shift of Cd_0.5_Cu_0.5-*x*_Ag_*x*_ Fe_2_O_4_ (**b**), and X-RD patterns of Cd_0.5_Cu_0.5-*x*_Ag_*x*_ Fe_2_O_4_, where *x* = 0.0 with JCPDS data base (**c**)

XRD patterns of Cd_0.5_Cu_0.5-*x*_Ag_*x*_ Fe_2_O_4_ ferrite powder synthesized by co-precipitation method are shown in Fig. 1. The peaks of all the synthesized samples were precisely indexed to the lattice planes (220), (311), (222), (400), (511), (440), (620), and (533), and there was no evidence of precursor impurity phases, proving the samples’ purity. Regarding JCPDS documents 591–0028 and 153–9598, the diffraction peaks match cubic spinel structure. The spinel phase’s development is shown by the strong peak visible at the (311) plane (Tanveer et al. 2022).

**Fig. 2:**
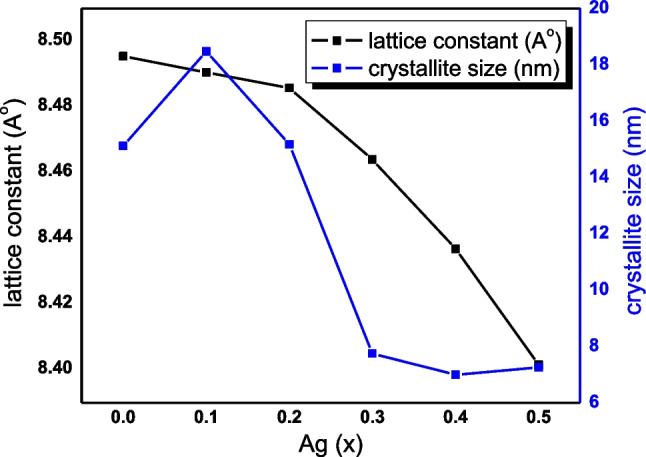
Lattice constant and crystallite size versus Ag^+^ ions content of Cd_0.5_Cu_0.5-*x*_Ag_*x*_ Fe_2_O_4_ samples

As the Ag^+^ content increased (i.e., when *x* was 0.4 and 0.5), the highest peaks slightly shifted toward lower angles, which confirm the replacement of Cu^2+^ with Ag^+^. Moreover, at high Ag^+^ content, the peaks broadened, confirming the formation of the ferrite nanostructure (El-hagary et al. 2013). Using the Debye–Scherrer equation, the average crystallite size (*D*) was calculated as follows, Eq. (3) (Hankare et al. 2012):3where *λ* = 1.542 Å and *θ* and *β* are the Bragg angle and full width at half maximum of the peak with the highest intensity (311). As the Ag^+^ content increased, the crystallite size fell from 15 to 7 nm, Fig. 2. This could be attributed to the difference of ionic radii of Ag^+^ (1.26) and Cu^2+^ (0.73), or the formation of secondary Ag complex phase. In addition, the lattice constant (*a*) was determined by Eq. (4) (Amin et al. 2020).

4 $${a}_{\mathrm{exp}}=d\sqrt{{h}^{2}+{k}^{2}+{l}^{2}}$$

where *d* is the inter-planar distance, which may be calculated using Bragg’s equation, and *h*, *k*, and *l* are the Miller indices (Amin et al. 2020),

5 $$d=\frac{n\uplambda }{2\;\mathrm{sin}\;\theta }$$

As shown in Table 1 and Fig. 2, the crystallite size ranged between 15 and 7 nm. Moreover, a value decreased as the Ag^+^ content increased, although the ionic radius of Ag^+^ is larger than that of Cu^2+^. The unit-cell volume was determined as *V* = $${a}^{3},$$ Table 1. The X-ray density (*d*_*x*_) was also derived from the XRD data as $${d}_{x}$$ =$$\frac{8M}{NV}$$, where *N* is the Avogadro number (6.0221 × 10^23^ g mol^−1^), *M* is the molecular weight of the produced materials, and *V* is the volume of its smallest cell (Ajaz et al. 2019; Junaid et al. 2019). Furthermore, the theoretical bulk density (*d*_*b*_) was also estimated from XRD data as $${d}_{b}$$ = $${m/\pi r}^{2}h$$, where *m* is mass, *r* is radius, and *h* is thickness of nanoparticles pallets, respectively (Coutinho and Verenkar 2019; Kale et al. 2018). Table 1 also includes the calculated relative density ($${d}_{R}\left(\%\right)$$= $${d}_{x}$$/$${d}_{b}$$), porosity (*P* = 1 − $$\frac{{d}_{b}}{{d}_{x}}$$ ), specific surface area (*S* = $$\frac{6000}{{d}_{x}D}$$), and dislocation density ($$\delta$$ =$$\frac{15\varepsilon }{aD}$$), where (*ε* = $$\frac{1}{{d}^{2}}$$). The polaron radius ($${\gamma }_{p}$$) was calculated as ( $${\gamma }_{p}$$ = $$\frac{1}{2}$$($$\sqrt[3]{\frac{\pi }{6{N}^{^{\prime}}}}$$)), where (*Nʹ* = 96/$${a}^{3})$$ (Amin et al. 2020; Ajaz et al. 2019).

**Table 1:** Nominal composition, 2*θ* of (311) peak, crystallite size (nm), average lattice constant (${a}_{\mathrm{exp}}$), unit cell volume (Å)^3^, d-spacing (Å), X-ray and bulk densities (${d}_{x}$ and${d}_{b}$) (g.${\mathrm{gm}}^{-3}$), relative density (${d}_{\mathrm{R}}$), porosity (*P*), polaron radius (${\gamma }_{\mathrm{p}}$) (Å), specific surface area (${\mathrm{m}}^{2}$ /g), and dislocation density (g/${\mathrm{m}}^{3}$) of Cd_0.5_Cu_0.5-*x*_Ag_*x*_ Fe_2_O_4_

Parameter	*x* = 0.0	0.1	0.2	0.3	0.4	0.5
2*θ* of (311) peak	35.00	35.00	35.00	34.50	34.50	34.50
d-spacing (Å)	2.56	2.56	2.56	2.55	2.54	2.53
Crystallite size (nm)	15.12	18.47	15.18	7.75	6.99	7.26
a (Å)	8.49	8.49	8.48	8.46	8.43	8.40
Volume of unit cell (Å)^3^	613.14	612.07	611.05	606.37	600.54	593.04
${d}_{x}$(g/cm^3^)	5.71	5.82	5.92	6.06	6.22	6.40
${d}_{\mathrm{b}}$(g/cm^3^)	3.010	3.045	3.110	3.180	3.210	3.260
${d}_{\mathrm{R}} ($%)	189.75	191.06	190.47	190.78	193.88	196.37
Porosity	0.473	0.477	0.475	0.476	0.484	0.490
Polaron radius (${\gamma }_{\mathrm{p}}$) (Å)	0.748	0.748	0.747	0.750	0.750	0.753
Dislocation density (g/cm^3^)	0.0178	0.0146	0.0178	0.0351	0.0393	0.0383
Specific surface area (m^2^/g)	69.47	55.83	66.73	127.56	137.83	129.11

#### FESEM results

**Fig. 3:**
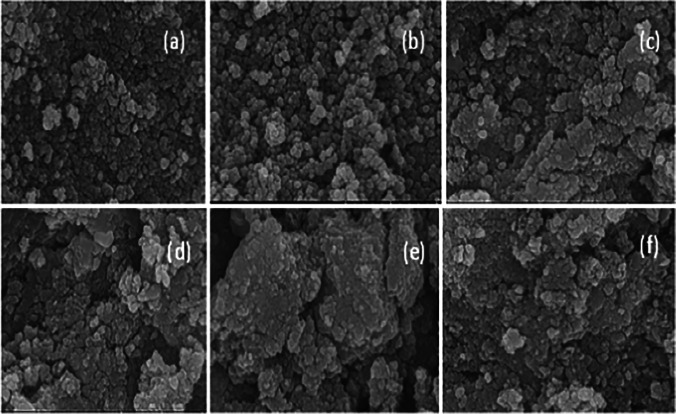
SEM images of Cd_0.5_ Cu _0.5-**x**_Ag_**x**_Fe_2_O_4_, **a** *x* = 0.0, **b** *x* = 0.1, **c** *x* = 0.2, **d** *x* = 0.3, **e*** x* = 0.4, and **f** *x* = 0.5

**Fig. 4:**
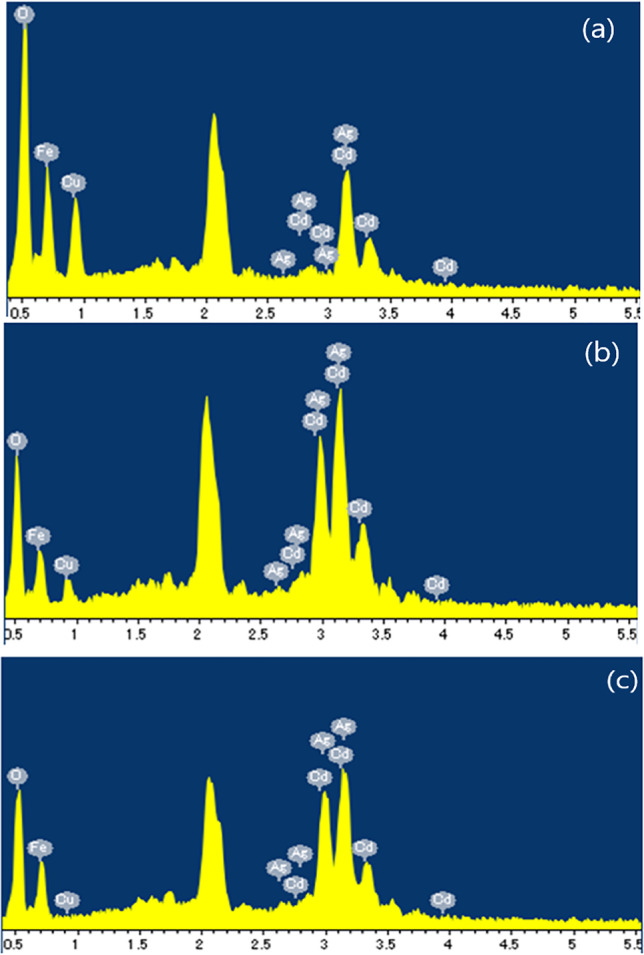
EDXs of Cd_0.5_Cu_0.5-*x*_Ag _*x*_Fe_2_O_4_, where **a** *x* = 0.0, **b** *x* = 0.3, and **c** *x* = 0.5

Figure 3 displays the FESEM images of the prepared nanoferrites. To various degrees, the compactly arranged nanoparticles consisted of agglomerated nanoparticles with various shapes, such as spherical, platelet, and prismatic-like (Berastegui et al. 2018). Their aggregation indicates that the magnetic nanoparticles in powder form had a strong mutual interaction (Gupta et al. 2015, 2018). The energy dispersive X-ray (EDX) spectra of the nanoferrites were acquired to determine their elemental composition. Figure 4, reveals the coexistence of Cd, Cu, Fe, Ag, and O without any contaminant, which indicates that the undesired components were completely removed. Table 2 shows an agreement between EDX results and the theoretical stoichiometry of the various elements in the samples.

**Table 2:** Compositional analysis for Cd_0.5_Cu_0.5-*x*_Ag_*x*_Fe_2_O_4_

Element (wt. %)	*x* = 0.0	*x* = 0.1	*x* = 0.2	*x* = 0.3	*x* = 0.4	*x* = 0.5
Fe	29.74	26.07	35.2	36.58	30.34	35.8
Cd	6.3	5.03	8.01	8.41	6.22	7.28
Cu	9.12	8.61	4.05	2.73	1.69	0.0
Ag	0.0	0.58	3.16	7,68	6.12	7.73
O	54.84	59.71	49.58	44.6	55.62	49.19

#### HR-TEM results

**Fig. 5:**
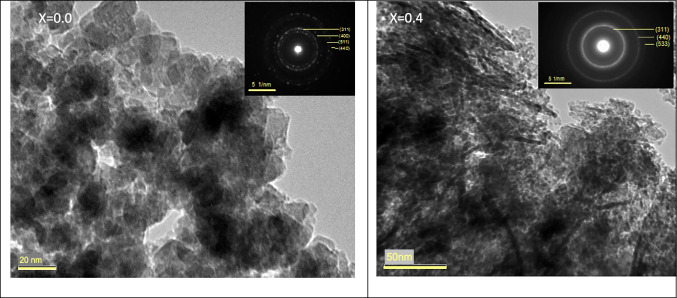
TEM images and selected area electron diffraction (SAED) of Cd_0.5_Cu_0.5-*x*_Ag_*x*_Fe_2_O_4_, **a** *x* = 0.0 and **b** *x* = 0.4

To investigate the morphology of spinel Cd_0.5_Cu_0.5−*x*_Ag_*x*_Fe_2_O_4_ nanoparticles, high-resolution transmission electron microscopy (HR-TEM) was used. The HR-TEM images of pure Cd_0.5_Cu_0.5_ Fe_2_O_4_, and Cd_0.5_Cu_0.1_Ag_0.4_Fe_2_O_4_ nanoparticles with average particle sizes below 50 nm and sphere-like shapes are shown in Fig. 5 (a), (b), respectively. The obvious and regular brilliant rings in the selected area electron diffraction (SAED) pattern of nanoparticles, Fig. 5, showed the ferrite’s high-crystalline quality; besides, the presence of patchy rings indicates that the polycrystalline nanomaterials are disseminated. This demonstrates that very crystalline natural nanoparticles were obtained (Kavitha and Kurian 2019; Mansour et al. 2018; Najmoddin et al. 2014). The inter-planar distances derived from the SAED rings, as well as the corresponding indexing sequence, matched those calculated from the XRD data rather well, revealing phase purity (spinel) of the synthesized materials.

#### Fourier-transform infrared (FT-IR) spectra

**Fig. 6:**
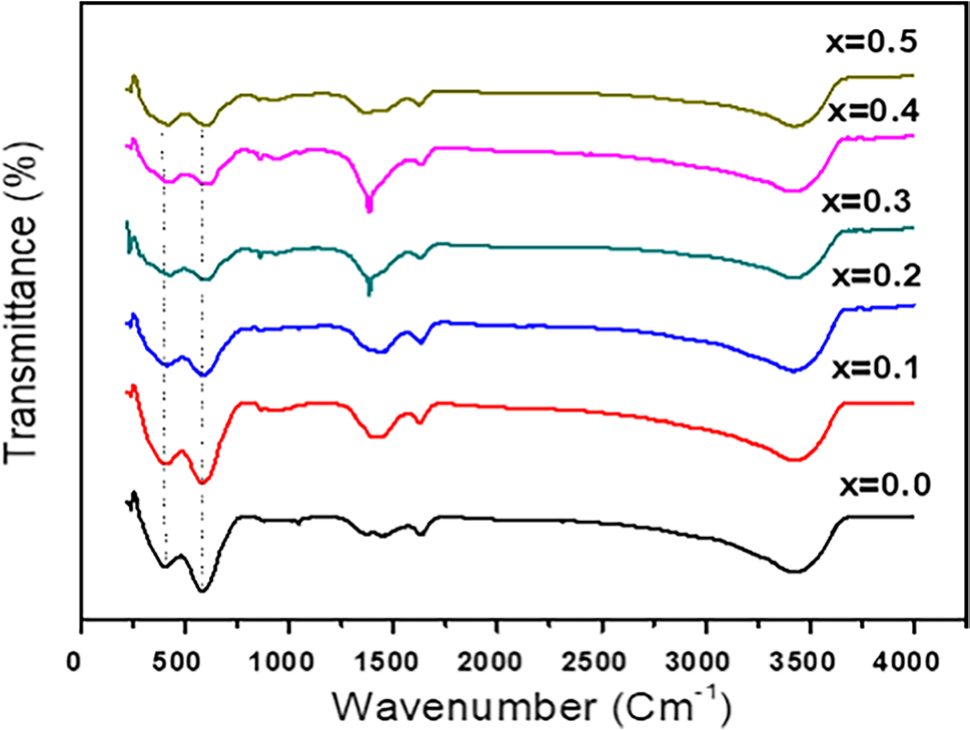
FT-IR spectra of Cd_0.5_Cu_0.5-*x*_ Ag_*x*_Fe_2_O_4_ spinel ferrite samples

FT-IR spectroscopy is a crucial method for examining the structural characteristics of ferrites and providing details on their phase. The spectra of the prepared samples are shown in Fig. 6, where two prominent absorption bands at 600 and 410 cm^−1^ are visible. These bands are attributed to the vibration of tetrahedral metal–oxygen bonds and octahedral metal–oxygen bonds, respectively (Abu-Elsaad and Abdel Hameed 2019). Moreover, starting from Ag^+^ contents (i.e., *x* ≥ 0.2), both the octahedral and tetrahedral site bands move toward higher wavenumbers due to the substitution of greater atomic weight Ag^+^ compared with Cu^2+^ (Ega et al. 2019). FT-IR measurements also revealed significant bands at 3450 cm^−1^ due to O–H groups and the samples’ development of cubic spinel phase is attributed to absorption bands in the region of 1300 cm^−1^ (Abu-Elsaad and Abdel Hameed 2019; Abdel Maksoud et al. 2020).

#### TGA results

**Fig. 7:**
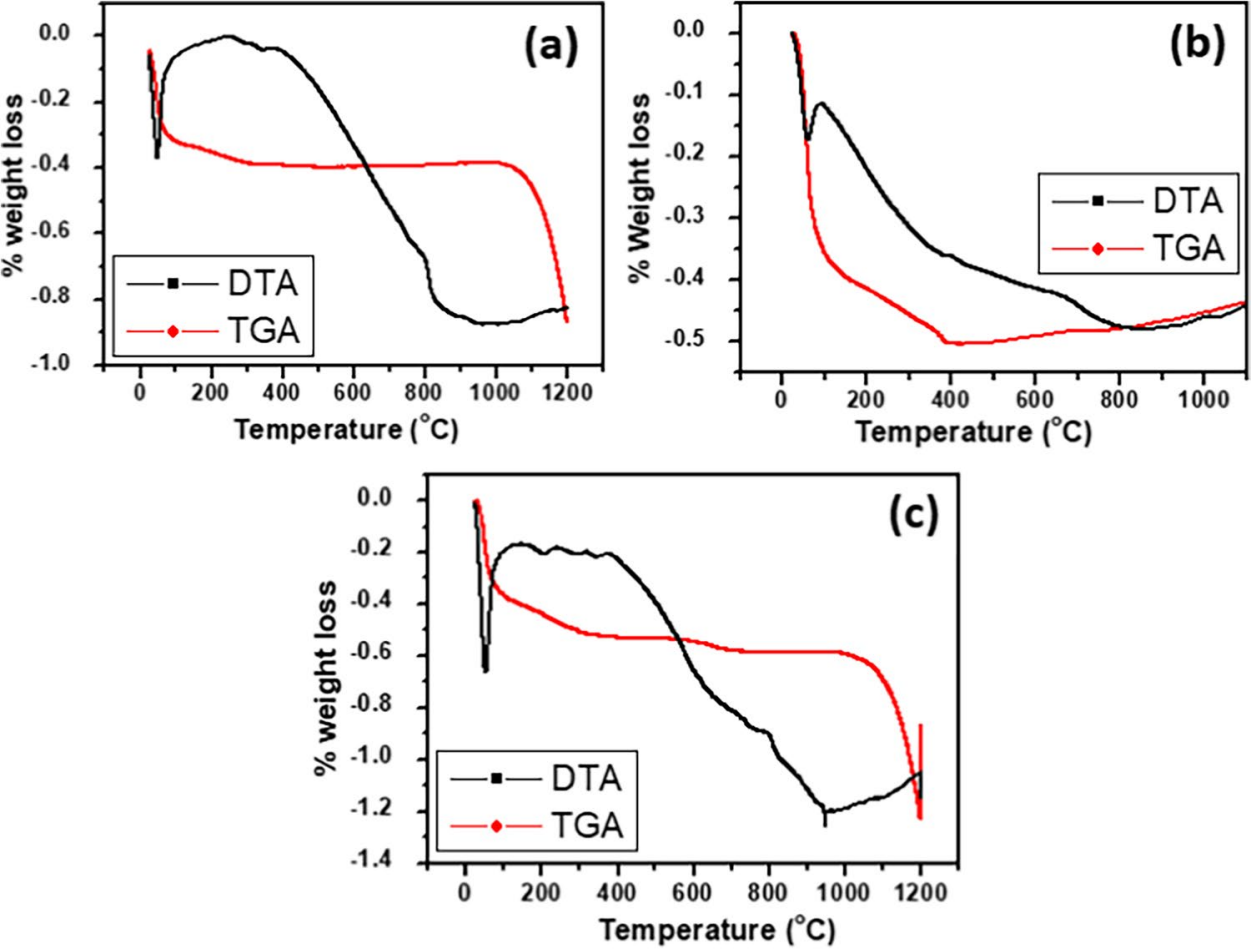
TGA and DTA curves of Cd _0.5_Cu_0.5-*x*_ Ag _*x*_ Fe_2_O_4_ where **a** *x* = 0.0, **b** *x* = 0.3, and **c** *x* = 0.5

The thermograms of the synthesized Cd_0.5_Cu_0.5−*x*_Ag_*x*_ Fe_2_O_4_ samples with different *x* values, Fig. 7, were measured to evaluate their thermal stability upon temperature levitation up to 1000 °C. The first weight loss before 100 °C could be ascribed to residual moisture loss, which is approved by the weak endothermic peak in the differential thermal analysis curve (Kharazi et al. 2019). The second weight loss above 200 °C could be assigned to the hydration water in the sample, which associated with the nanoparticle dislocations to attain a stable configuration. Around 800 °C, the third weight loss may have resulted from the oxidation of the corresponding transition metal hydroxides giving rise to the metal oxides. Above 800 °C, the crystallization process took place with the formation of nanogranular-type ferrites (Satyanarayana et al. 2019).

#### Magnetic properties

**Fig. 8:**
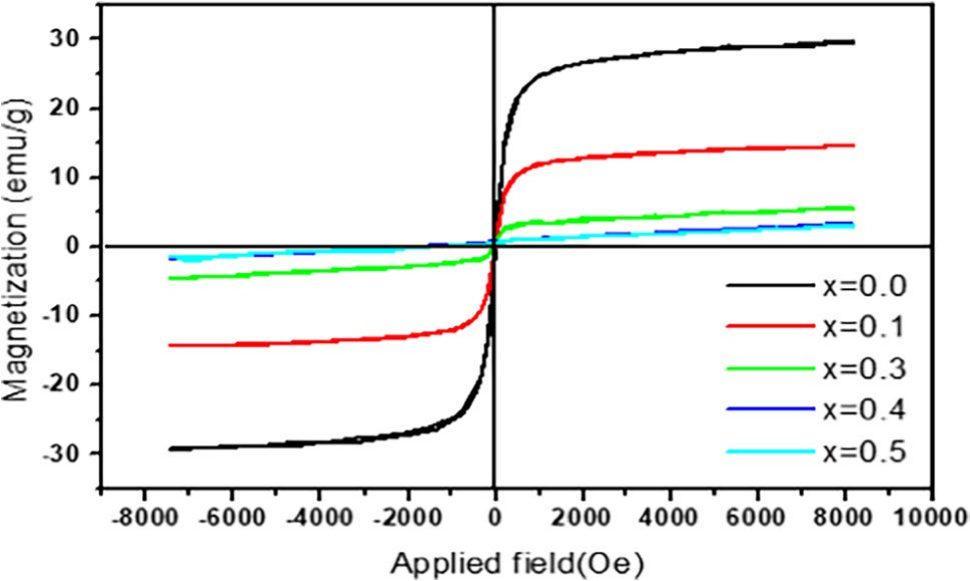
Hysteresis loop of Cd_0.5_Cu_0.5-*x*_Ag_*x*_Fe_2_O_4_ ferrite samples

The magnetic properties of the Cd_0.5_Cu_0.5−*x*_Ag_*x*_Fe_2_O_4_ samples were examined with a vibrating-sample magnetometer at room temperature. Figure 8 displays the resulting S-type curves of the *M*–*H* loops. The magnetization curves showed no hysteresis loop, demonstrating the paramagnetic characteristics of the synthesized materials. The magnetic properties of nanoferrites could result from various factors, including the fabrication technique, particle size, and cation exchange at the lattice sites. *M*_*S*_ dropped from 29.33 to 2.80 emu/g when the Ag^+^ content increased.

This may be explained by the cation exchange inside the crystallite sites of the Ag^+^-substituted nanoferrites. By relocating Ag^+^ to the B sites and removing Cu^2+^ from the A sites, the A–B superexchange interactions were suppressed. The saturation magnetization diminished with increasing the Ag^+^ content, perhaps due to its nonmagnetic nature (Berastegui et al. 2018; Gholizadeh and Jafari 2017). Furthermore, the *M*_*S*_ value of a porous nanocrystalline magnetic material is generally determined by its porosity as well as the form and size of the holes in it; this explains why, as listed in Table 1, the porosity increased linearly with the Ag^+^ substitution, representing another cause for the *M*_s_ decrease.

#### Raman spectra

**Fig. 9:**
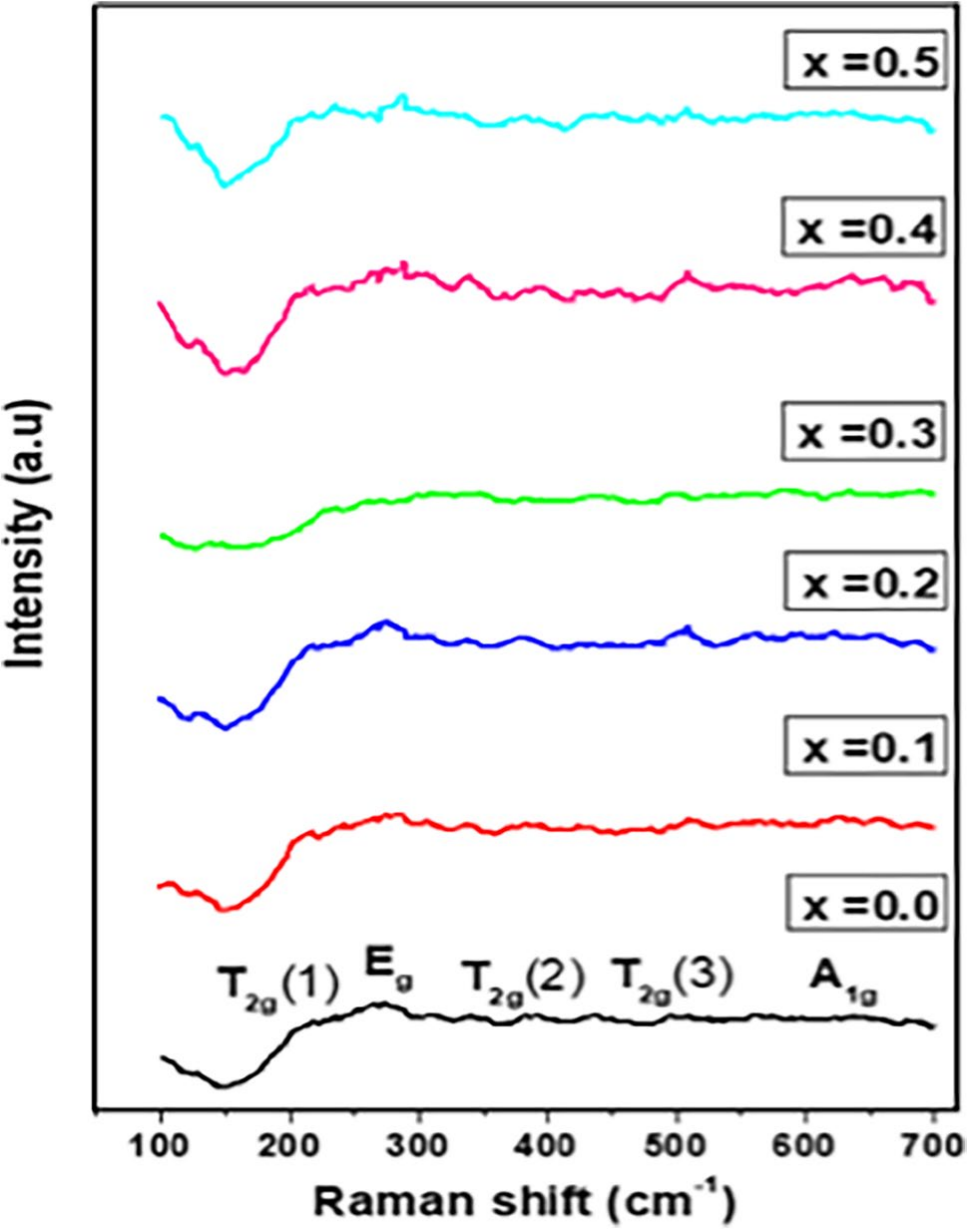
Raman spectroscopy of Cd_0.5_Cu_0.5-*x*_Ag_*x*_Fe_2_O_4_ ferrite samples

Raman spectroscopy is an effective technique to examine the lattice effects brought on by vibrational modes. As shown in Fig. 9, there are five active modes: A_1g_, E_g_, and three T_2g_ (Aslam et al. 2021). The three T2g modes, T2g(1), the lowest frequency mode, T2g(2), and T2g(3), the highest frequency modes of this vibrational species are designated. The development of the A_1g_, E_g_, and 3T_2g_ Raman active peaks were predicted to occur from the formation of the cubic phase, which is clearly seen in the spectra of all prepared samples.

Raman peak frequencies over 600 cm^−1^ indicate the tetrahedral site, while those below 600 cm^−1^ indicate the octahedral site in the case of spinel ferrite (Andhare et al. 2021). However, the five Raman modes could be attributed to the migration of O_2_ − anions and cations at tetrahedral and octahedral sites in spinel ferrites. The O_2_^−^ anion is visible in the A1g Raman mode, and the O_2_ and cations are seen in the motion of the remaining four Raman modes (Eg and three T2g) (Aslam et al. 2021). Stretching of the M–O bond results in the T2g(1) mode at around 210 cm^−1^. Due to the mobility of metal ions with four oxygen atoms in the octahedral sites, T2g(2) modes appear at about 391 cm^−1^. In addition, the M–O symmetric bending at the octahedral sublattice causes the Eg mode appears at about 270 cm^−1^. Also, the M–O symmetric stretching at the tetrahedral plane causes the A1g(1) mode appeared at about 633 cm^−1^ (Das et al. 2019).

### Kinetics studies

**Fig. 10:**
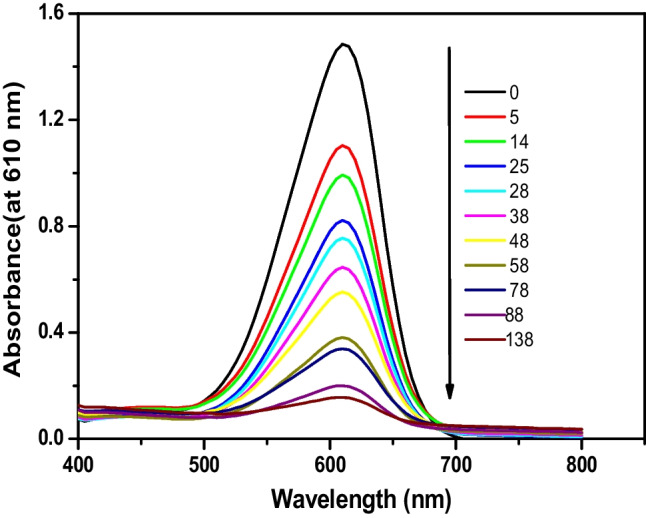
Absorbance of IC dye vs time plot for [IC] = 10^−4^ mol l^−1^, [H_2_O_2_] = 0.079 mol l^−1^ and in the presence of 10 mg of Cd_0.5_Cu_0.5−*x*_Ag_*x*_Fe_2_O_4_ at 30 °C

**Fig. 11:**
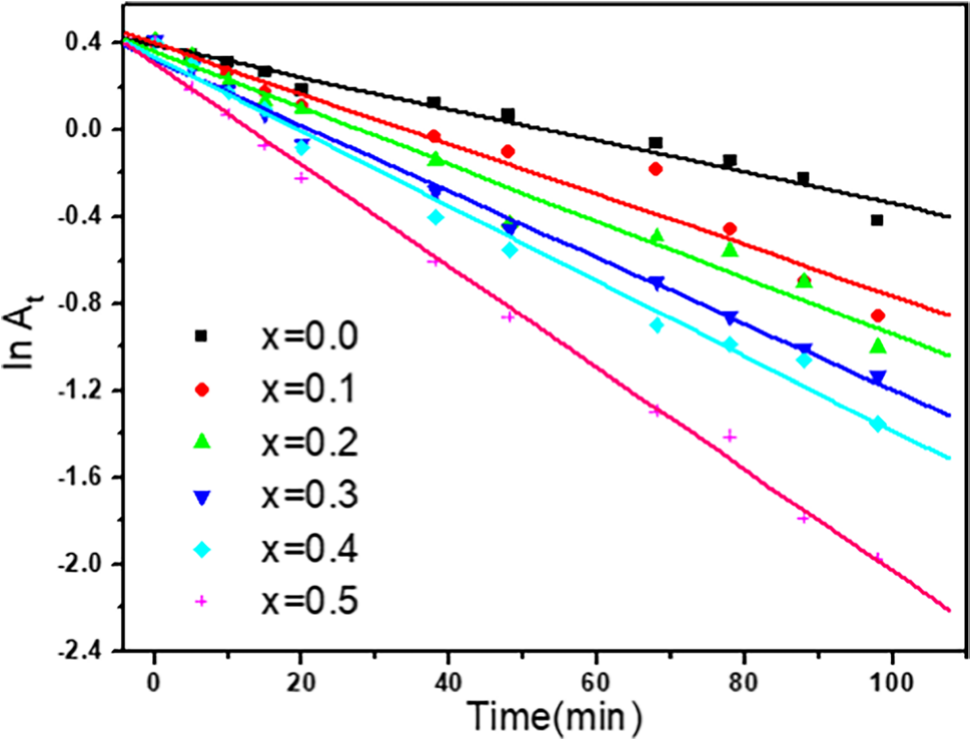
Pseudo first-order reaction of oxidation of IC = 10^−4^ mol l^−1^, H_2_O_2_ = 0.079 mol l^−1^, and 10 mg of Cd_0.5_Cu_0.5−*x*_Ag_*x*_Fe_2_O_4_ at 30 °C

The catalytic capacities of the synthesized Ag^+^-substituted nanoferrites through the oxidative degradation of IC under various experimental settings by using H_2_O_2_ as an oxidant were assessed. The factors such as H_2_O_2_ and IC concentrations, catalyst quantity, temperature, pH, reaction duration, and catalyst reusability affecting the degradation process were considered. When IC was combined with H_2_O_2_ in absence of the catalyst, no absorbance change was detected. In contract, when the catalyst was added to this combination, however, the IC absorbance gradually decreased (Fig. 10). The reaction was considered to be pseudo-first-order, and the rate constant, *k*, was derived using the slope of $\mathrm{ln}\frac{{A}_{o}}{({A}_{o}-{A}_{t})}=kt$, where *A*_o_ and *A*_*t*_ are the absorbance at time 0 and *t* (in minutes), respectively, as observed in Fig. 11. The calculated parameter values are listed in Table 3.

**Table 3:** Effect of the prepared samples as catalyst on the rate of oxidation with 10 mg of each samples. [H_2_O_2_]_o_ = 0.079 mol l^−1^ and [IC]_o_ = 10^−4^ mol l^−1^ at 30 °C

Cd_0.5_Cu_0.5-*x*_Ag_*x*_Fe_2_O_4_ catalyst	${k}_{\mathrm{obs}}$× 10 ^−2^ (min^−1^)	*R*^2^
*x* = 0.0	0.73	0.9869
*x* = 0.1	1.161	0.97837
*x* = 0.2	1.306	0.9867
*x* = 0.3	1.525	0.99616
*x* = 0.4	1.719	0.99429
*x* = 0.5	2.338	0.99788

#### Effect of catalyst dosage

At IC and H_2_O_2_ concentrations of 10^−4^ mol L^−1^ and 0.079 mol L^−1^, respectively, the catalyst dosage was varied (5, 10, 15, 20, and 25 mg) to examine its effect on the IC removal. As the catalyst dosage increased from 5 to 25 mg, the rate constant increased from 0.011 to 0.039 min^−1^, which indicates that more active sites became accessible for H_2_O_2_ activation and production of an intermediate active species, allowing further and quicker IC molecule degradation (Hassani et al. 2018).

#### H_2_O_2_concentration effects

**Fig. 12:**
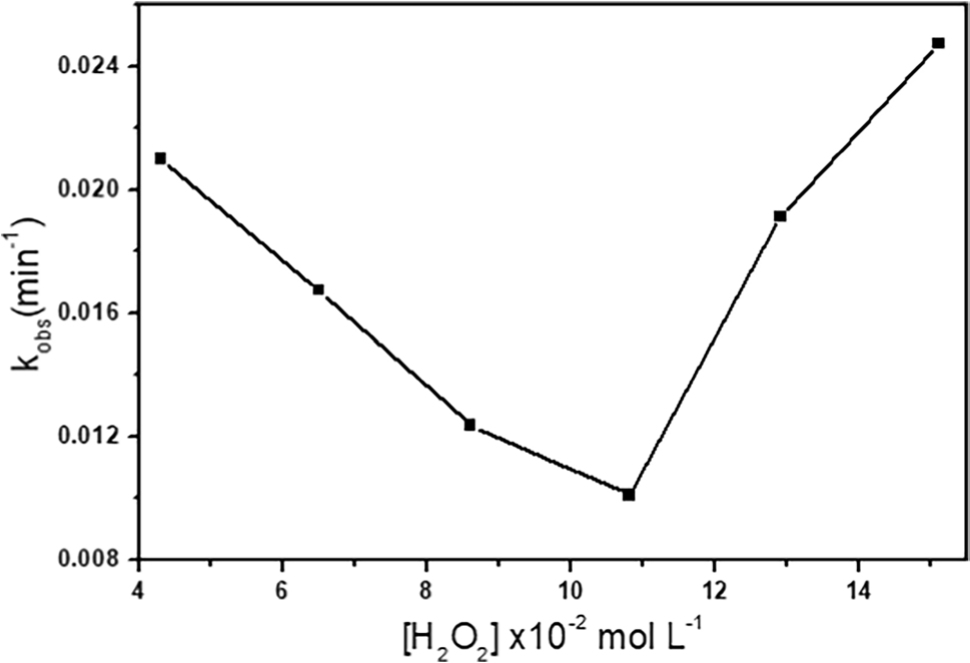
Dependence of the rate constant on [H_2_O_2_]_o_, [IC]_o_ = 10^−4^ mol l^−1^ with 10 mg of Cd_0.5_Cu_0.5−*x*_Ag_*x*_Fe_2_O_4_ (where *x* = 0.5) at 30 °C

The concentration of H_2_O_2_ varied from 0.039 to 0.139 mol L^−1^, while the initial IC concentration and the other parameters were kept constant to study its effect. As shown in Fig. 12, the reaction rate decreased when the concentration of H_2_O_2_ was raised from 0.039 to 0.099 mol L^−1^ then it increased when further incrementing this parameter, indicating that an excess of H_2_O_2_ reduces the ^•^OH scavenging action. The hydroxyl radical reacted with the excess of H_2_O_2_ to create the perhydroxyl radical, which is less oxidizing than ^•^OH, and hence reduced the IC oxidation rate (Abo-Farha 2010; Chanderia et al. 2017; Wu et al. 2016). Increasing the H_2_O_2_ concentrations over 0.099 mol L^−1^ might have caused HO_2_^•^ recombination to create H_2_O_2_, which then decomposed to HO^•^, increasing the reaction rate.

#### Effect of IC concentration

**Fig. 13:**
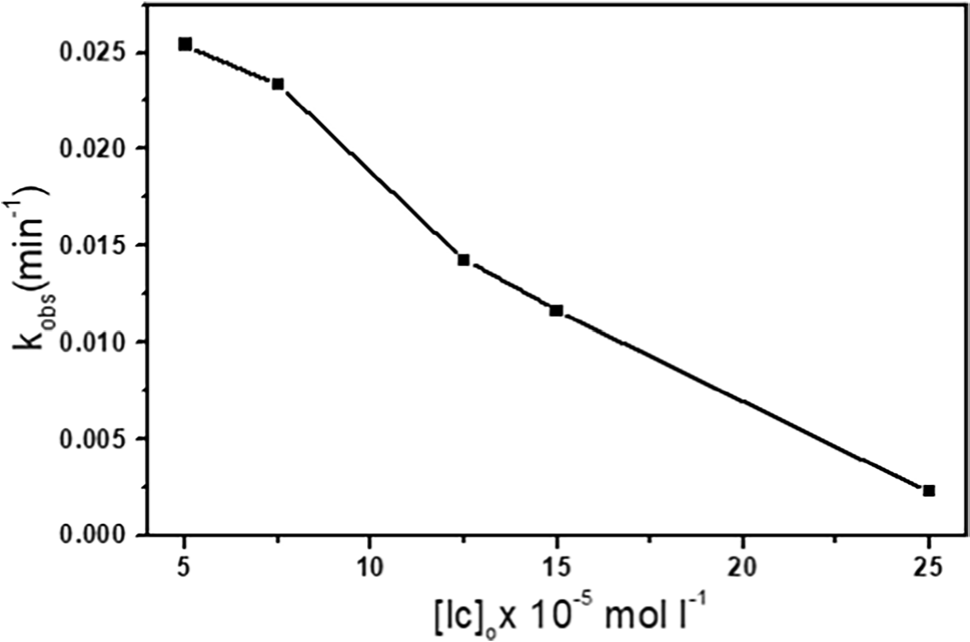
Dependence of the rate constant on ${[IC]}_{o}$ with H_2_O_2_ = 0.079 mol l^−1^ and 10 mg of Cd_0.5_Cu_0.5−*x*_Ag_*x*_Fe_2_O_4_ (where *x* = 0.5) at 30 °C

The IC concentration effect on the IC oxidation was also investigated in the range between 5 × 10^−5^ and 25 × 10^−5^ mol L^−1^ at constant H_2_O_2_ concentration and catalyst dosage. With an increase in IC concentration, the reaction rate slowed, Fig. 13. This pattern may be explained by the fact that there are very few attacking active species involved in the degrading process. (Gemeay et al. 2017). IC molecules may also assemble in the catalytic sites at high concentrations. As a result, the slowed interaction between H_2_O_2_ and catalyst resulted in a barrier to the generation of free radicals. The observed reduction in the IC oxidation rate when increasing the dye concentration indicates that the process followed first-order kinetics (Hosseini-Zori and Mokhtari Shourijeh 2018).

#### Effect of pH

**Fig. 14:**
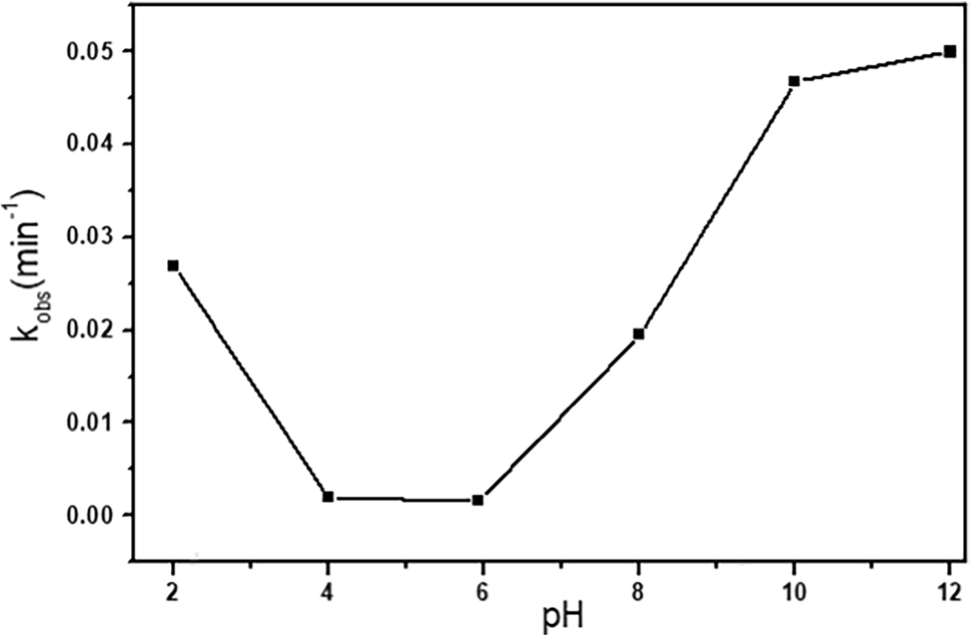
Dependence of the rate constant on pH with[IC]_o_ = 10^−4^ mol l^−1^, H_2_O_2_ = 0.079 mol l^−1^, and 10 mg of Cd_0.5_Cu_0.5−*x*_Ag_*x*_Fe_2_O_4_ (where *x* = 0.5) at 30 °C

The rate of IC oxidation by H_2_O_2_ catalyzed by Cd_0.5_Ag_0.5_ Fe_2_O_4_ ferrite was measured at various pHs. Phosphate buffer (KH_2_PO_4_/Na_2_HPO_4_) with a pH range of 4.8 to 8 was used to modify the original pH of the IC solution. Other desirable pH values outside of the phosphate buffer working range were changed by adding 0.1 mol l^−l^ HCl or NaOH aliquots. Figure 14 depicts the fluctuation of the rate constant with pH. It is obvious that the reaction had a moderate rate in a strongly acidic medium, the lowest rate in weak acidic, and a progressive increase in neutral weak alkaline media before reaching the greatest rates in alkaline solution. The pKa value of IC was measured to be 11.17, whereas the pKa of H_2_O_2_ is 11.6 and is known from the literature (Moiseev 1997). When it comes to the Fenton process, most papers recommend using an acidic media to boost oxidation efficiency (Choi and Bokare 2014). The generation of the HO_2_• radical at an initiation step with the Fe^+3^/H_2_O_2_, Eqs. (6) and (7), which is a less reactive radical than the HO• radical, could be explained by the tiny difference in the rate constant drop. The hydroxyl radical is also a non-selective oxidant that damages organic molecules via hydrogen abstraction and hydroxyl addiction (Kilic et al. 2008).

6 $${\mathrm{Fe}}^{3+}+{\mathrm{H}}_{2}{\mathrm{O}}_{2}\to {\mathrm{Fe}}^{2+}+\mathrm{H}{{\mathrm{O}}_{2}}^{\bullet }+{\mathrm{H}}^{+}$$

7 $${\mathrm{Fe}}^{2+}+{\mathrm{H}}_{2}{\mathrm{O}}_{2}\to {\mathrm{Fe}}^{3+}\cdots +{\mathrm{HO}}^{\bullet }+{\mathrm{HO}}^{-}$$

Despite its reputation as an oxidant, H_2_O_2_ is an effective reducing agent under alkaline circumstances, which means that the Ag^+^ ions in ferrite nanoparticles could be reduced to zero-valent Ag^0^ (Gatemala et al. 2015). The turnover frequency of Ag^o^ during the catalyzed decomposition of H_2_O_2_ has been shown to be highly impacted by pH, ranging from 1776.0 at pH 11.0 to 3.2 min^−1^ at pH 3.0 (He et al. 2012). The rate constant determined over the pH range can also be explained by the variation in the speciation of H_2_O_2_ with pH. The redox reaction of the substituted Ag^+^ in the lattice structure of site A and/or on the surface of ferrites nanoparticles with H_2_O_2_, which produced an intermediate radical species and led to the oxidative degradation of IC dye, could therefore be responsible for the increase in reaction rate under alkaline conditions Eq. (8)–(10),

8 $$\begin{array}{cc}{\mathrm{H}}_{2}{\mathrm{O}}_{2}+2{\mathrm{HO}}^{-}\to 2{{\mathrm{H}}_{2}\mathrm{O}+{\mathrm{O}}_{2}+2\mathrm{e}}^{-}& \left({E}^{0}ox=0.146 \mathrm{V}\right)\end{array}$$

9 $$\begin{array}{cc}{\mathrm{H}}_{2}{\mathrm{O}}_{2}+2{\mathrm{Ag}}^{+}+2{\mathrm{HO}}^{-}\to 2{\mathrm{Ag}}^{0}+{2\mathrm{H}}_{2}\mathrm{O}+{\mathrm{O}}_{2}& \left({E}^{0}=0.947 \mathrm{V}\right)\end{array}$$

10 $$\begin{array}{cc}2{\mathrm{Ag}}^{0}+{\mathrm{H}}_{2}{\mathrm{O}}_{2}\to 2{\mathrm{Ag}}^{+}\cdots +2{\mathrm{HO}}^{\bullet }& \left({E}^{0}=0.068 \mathrm{V}\right)\end{array}$$

What is noteworthy is that the Cd_0.5_Cu_0.5-*x*_Ag_*x*_Fe_2_O_4_ exhibited excellent catalytic performance in the whole pH range of 2–11, especially in the strong alkaline environment.

#### Effect of Ag^+^ ion doping

**Fig. 15:**
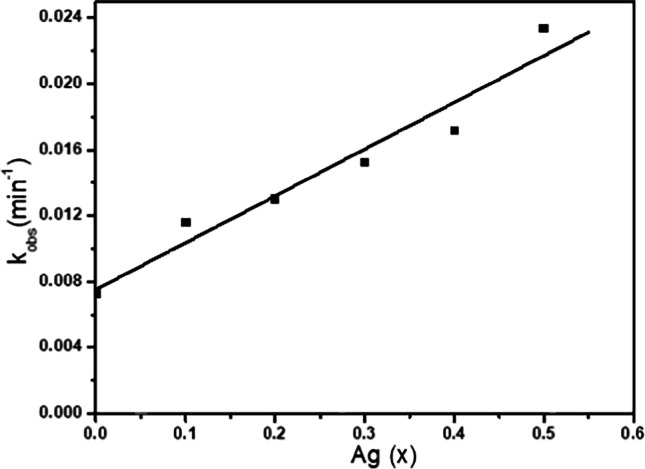
Relationship between Ag concentration (*x*) and rate constant of Cd_0.5_Cu_0.5−*x*_Ag_*x*_Fe_2_O_4_ (where *x* = 0.5) at 30 °C

**Fig. 16:**
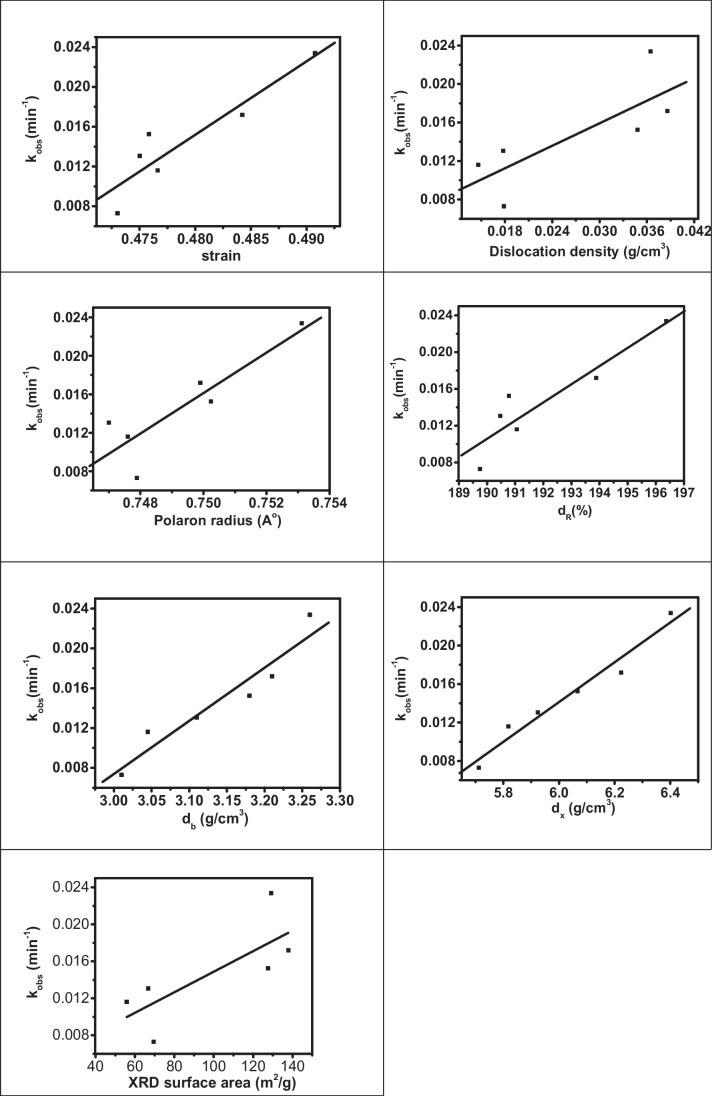
Relation between X-ray variables and rate constant of spinel ferrites

Exchanging a small fraction of spinel ferrites by dopants is an effective strategy to improve catalytic performance. The synergistic catalytic effect of the Cd_0.5_Cu_0.5-*x*_Ag_*x*_Fe_2_O_4_ NPs was evaluated in the model reaction of IC degradation using H_2_O_2_ as an eco-friendly oxidant. A complete decolorization within 90 min with 90% degradation of 10^−4^ mol l^−1^ IC solution with rate constant 0.0233 min^−1^ was obtained. As shown in Fig. 15, the rate constant increased from 0.0073 in the presence Cd_0.5_Cu_0.5_Fe_2_O_4_ NPs (Ag^+^  = zero) to 0.0233 min^−1^ in the presence of Cd_0.5_Ag_0.5_Fe_2_O_4_ (Cu^2+^  = 0). This means that the insertion of Ag^+^ promoted the rate constant more than three times. It has been reported that Ag^+^/Ag^0^ mediated generation of reactive oxygen species (ROS), including O_2_^•−^ and ^•^OH (Di He et al. 2010; He et al. 2012). In addition, the catalysis process involves three basic redox couples Ag^+^/Ag^0^, Cu^2+^/Cu^+^, and Fe^3+^/Fe^2+^ (Moreno-Castilla et al. 2019). Moreover, Fe^3+^ can be reduced to Fe^2+^ by Cu^+^ because the redox potential of Cu^2+^/Cu^+^ (0.17 eV) is less than that of Fe^+3^/Fe^+2^ (0.771 eV) (Chen et al. 2020). The strong synergistic effect between these metals promoted the H_2_O_2_ activation. Ag^+^ and Cu^2+^ function as the synergistic coactive sites to catalyze the IC degradation, which appears to be the cause of the high activity seen, while Fe^3+^ serves to maintain the integrity of the spinel structure, which may be a factor in the catalyst’s amazing durability. To obtain a deeper understanding of the extent of the relationship between the catalytic activity of the ferrite samples doped with silver ions and their structural properties, the relationship between many of these variables and the rate constant values of the reaction has been drawn as shown in Fig. 16. Certainly, the figure proves that the reaction rate rises directly with raising the values ​​of all these variables.

#### Effect of temperature

**Fig. 17:**
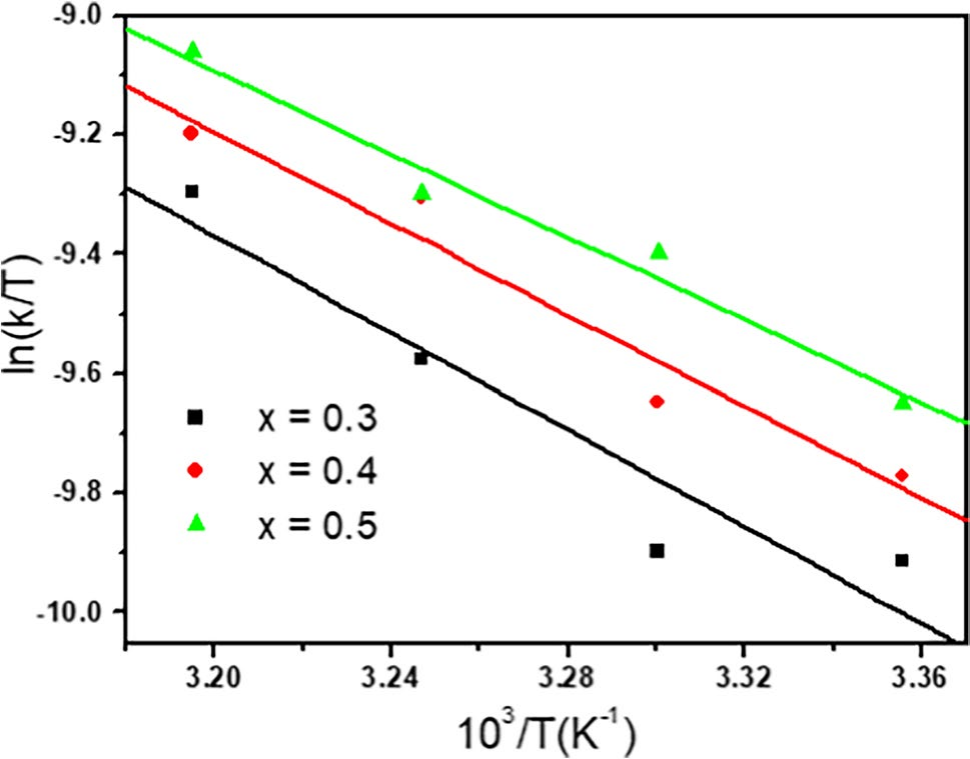
Eyring plot of for catalytic oxidation of [IC] = 10^−4^ mol/L with [H_2_O_2_] = 0.079 mol l^−1^ in the presence of 10 mg of Cd_0.5_Cu_0.5−*x*_Ag_*x*_Fe_2_O_4_ ferrite catalysts

The temperature range used for the IC oxidation was 25 to 40 °C. When the temperature rises, the reaction rate also increased due to generation of higher number of activated species necessary for IC oxidation. Hence, these species would increase the frequency of molecular collisions on the catalyst surface (Qu et al. 2014; Wu et al. 2016). The thermodynamic activation parameters, that is, enthalpy (*ΔH*^*#*^) and entropy (*ΔS*^*#*^), are derived from the slope and intercept of the Eyring plot, Eq. (11), Fig. 17.

11 $$\mathrm{ln}\frac{k}{T}=\boldsymbol{ }\frac{{\Delta H}^{\#}}{RT}+\mathrm{ln}\frac{{K}_{B}}{h}+\frac{{\Delta S}^{\#}}{R}$$

where *h* equal to the Plank constant (6.626 × 10^−34^ Js), *K*_*B*_ equal to the Boltzmann constant (1.38 × 10^−23^ JK), and *R* is the gas constant (8.31 J mol^−1^ K^−1^). The activation energy (*E*_*a*_) and free energy (*ΔG*^*#*^) were calculated, Eqs. (12) and (13).

12 $${E}_{a}=\Delta {H}^{\#}+R{T}_{\mathrm{exp}}$$

13 $$\Delta {G}^{\#}=\Delta {H}^{\#}-T\Delta {S}^{\#}$$

where *T*_exp_ is the average experimental temperature. The results are listed in Table 4. The positive *ΔG*^*#*^ values indicate the nonspontaneous nature of the reaction, whereas the positive *ΔH*^*#*^ and negative *ΔS*^*#*^ suggest that the reaction under study is endothermic with decrease in the degree of randomness (Gemeay et al. 2017; Harrache et al. 2019).

**Table 4:** The rate constant and the activation parameter for the oxidation of [IC] = 10^−4^ mol l^−1^ with [H_2_O_2_] = 0.079 mol l^−1^ and 10 mg of Cd _0.5_Cu _0.5-*x*_Ag_*x*_Fe_2_O_4_ catalyst

Catalyst	Temp, °C	*k* × 10^−2^, min^−1^	${E}_{a}$, kJ mol^−1^	Δ*H*^#^, kJ mol^−1^	Δ*G*^#^, kJ mol^−1^	Δ*S*^#^, J mol^−1^ K^−1^
*x* = 0.3	25	1.48	36.157	33.62	84.94	− 167.72
	30	1.52				
	35	2.13				
	40	2.87				
*x* = 0.4	25	1.7	34.44	31.904	84.38	− 171.76
	30	1.95				
	35	2.8				
	40	3.16				
*x* = 0.5	25	1.92	31.45	28.91	81.456	− 127.17
	30	2.51				
	35	2.83				
	40	3.63				

#### Recyclability

**Fig. 18:**
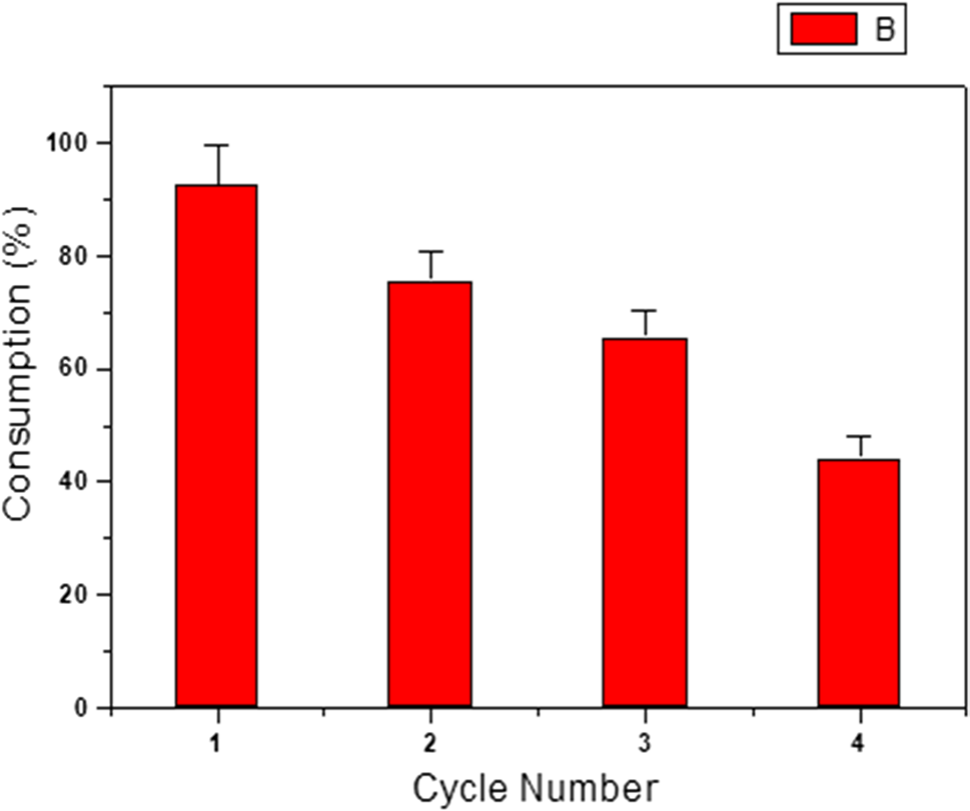
Recycling studies for the oxidation of [IC] = 10^−4^ mol Ll^−1^ with H_2_O_2_ = [0.079] mol l^−1^ and 10 mg of Cd_0.5_Cu_0.5−*x*_Ag_*x*_Fe_2_O_4_ (where *x* = 0.5) at 30 °C

The reusability test is very important to assess the stability and economic feasibility of the catalyst in the industrial applications. After each experimental study, the utilized catalyst was removed from the working environment and put through a series of cleaning and drying procedures before being used once more. With the aid of an external magnet, Cd_0.5_Cu_0.5−*x*_Ag_*x*_Fe_2_O_4_ was recycled four times from the reaction media. Figure 18 illustrates the reusability of the synthesized Cd_0.5_Cu_0.5−*x*_Ag_*x*_Fe_2_O_4_ over four cycles. The gradual exhaustion of the catalyst could be attributed to partial distortion of the crystal lattice of the catalyst due to the synergistic effects of metal ions might lead to the transformation of metal ions into the reduced form. The expected contribution of Fe^3+^ ions in the catalytic process diminished the stability of the crystal lattice. Moreover, the leaching of some substituted Ag^+^ will reduce the availability of catalyst active sites.

#### Reaction mechanism

The catalytic action of Fe^3+^, Cu^2+^, and Ag^+^ metal ions is involved in a typical reaction scheme for the degradation of IC using H_2_O_2_ catalyzed by ferrite samples. The cation distribution was the most important factor impacting the catalytic activity of the nanoferrites, as the ions at the B sites acted as active catalysts, but the cations at the A sites were catalytically inert. This means the catalytic activity would be increased as the Ag^+^ content increased owing to the B sites’ higher Ag^+^ content. The occupying of Cu^2+^ in the suboctahedral sites may also contribute to the catalytic activity. Therefore, Ag^+^ and Cu^2+^ work as synergistic coactive sites to catalyze IC breakdown, which appears to be the case, while Fe helps to maintain the spinel structure’s integrity, possibly contributing to the catalyst’s endurance. In general, the combination of the Fe^3+^, Cu^2+^, and Ag^+^ metal ions can have a modulating effect to increase the catalytic activity. Density functional theory calculations revealed that Fe^3+^/Cu^2+^ is the major active site, where electrons transfer from Cu^2+^ to Fe^3+^ ensures the low-valence state Fe^2+^ for catalytic oxidation (Wu et al. 2022). Reactive oxygen species (ROS), HO•, HO^2−•^, and O^2−•^ are oxidants found in the route that are produced as a result of the interaction of Fe^3+^, Cu^2+^, and Ag^+^ with H_2_O_2_ and surface hydroxyl groups. According to reports, the number of surface OH sites is influenced by the number of oxygen vacancy sites (Bonapasta et al. 2009; Namai and Matsuoka 2005). As a result, during the selective oxidation of methane over copper iron pyrophosphate catalysts, Cu^2+^ would oxidase the lattice oxygen to O_2_^•−^ while also being reduced to Cu^+^ (Polniˇsera et al. 2011). The involvement of free radical species in the reaction mechanism of H_2_O_2_ with metal ions was confirmed earlier by ESR spectroscopy using spin trapping (Khmelenko and Frolova 2021; Sharma et al. 2015; Zhang et al. 2022). In order to validate the production of the radical species, the radical scavengers tert-butanol and the chromogen 2, 2-azino-bis(3-ethylbenzthiazoline)-6-sulfate diammonium salt were utilized as probes (Gemeay et al. 2003). The following process is hypothesized based on these experimental findings and debate, and it indicates an interaction between H_2_O_2_ and the metal ions with the generation of extremely energetic ROS.

14 $${\mathrm{H}}_{2}{\mathrm{O}}_{2}\leftrightarrows \mathrm{H}{{\mathrm{O}}_{2}}^{-}+{\mathrm{H}}^{+}$$

15 $$\equiv {\mathrm{Cu}}^{2+}+\mathrm{H}{{\mathrm{O}}_{2}}^{-}\to \equiv {\mathrm{Cu}}^{+}+\mathrm{H}{{\mathrm{O}}_{2}}^{\bullet }$$

16 $$\equiv {\mathrm{Cu}}^{+}+{\mathrm{H}}_{2}{\mathrm{O}}_{2}\to \equiv {\mathrm{Cu}}^{2+}+{\mathrm{HO}}^{\bullet }+{\mathrm{HO}}^{-}$$

17 $$\equiv {2\mathrm{Ag}}^{+}+{\mathrm{H}}_{2}{\mathrm{O}}_{2} +2{\mathrm{HO}}^{-}\to {2\mathrm{Ag}}^{0}+{2\mathrm{H}}_{2}\mathrm{O}+{\mathrm{O}}_{2}$$

18 $$\equiv {\mathrm{Ag}}^{0}+{\mathrm{H}}_{2}{\mathrm{O}}_{2} \to 2 {\mathrm{Ag}}^{+}+2{\mathrm{HO}}^{\bullet }$$

19 $$\equiv {\mathrm{Fe}}^{3+}+\mathrm{H}{{\mathrm{O}}_{2}}^{\bullet } \to \equiv {\mathrm{Fe}}^{2+}+{\mathrm{O}}_{2}$$

20 $$\equiv {\mathrm{Fe}}^{3+}+{\mathrm{Cu}}^{+} \to \equiv {\mathrm{Fe}}^{2+}+{\mathrm{Cu}}^{2+}$$

21 $$\mathrm{H}{{\mathrm{O}}_{2}}^{\bullet }+{\mathrm{H}}_{2}{\mathrm{O}}_{2} \to {\mathrm{HO}}^{\bullet }+{\mathrm{O}}_{2}{+\mathrm{H}}_{2}\mathrm{O}$$

Thus, the formed ROS attacks the IC forming active intermediate, which then decomposes in the rate-determining step, giving the final oxidation products. This mechanism supports the rate enhancement on going to more alkaline conditions due to the dependence of the redox potential of the couple HO_2_^•^ (O_2_^• −^)/H_2_O_2_ (HO_2_^−^) on pH. The redox potential drops from 1.4 to 0.18 V over the pH range 0–14 (Luo et al. 1988).

22 $$\mathrm{IC}+\mathrm{ROS}\to \mathrm{intermediate\; compounds}\to \mathrm{final\; degradation\; products}$$

## Conclusions

Cd_0.5_Cu_0.5−*x*_Ag_*x*_Fe_2_O_4_ nanoferrites were synthesized via a simple co-precipitation method. XRD analysis revealed that as the Ag^+^ content increased, a secondary Ag complex phase appeared after *x* = 0.2. The shrinkage of the spinel unit cell was observed when increasing the Ag^+^. The lattice parameter value decreased from 8.4954 Å at *x* = 0 to 8.4015 Å at *x* = 0.5. Ag^+^ and Cu^2+^ were distributed between the tetrahedral and octahedral sites in the spinel lattice. The magnetization curves of the prepared samples *x* = 0.0, 0.1, 0.2, and 0.3 showed a hysteresis loop, demonstrating the ferromagnetic characteristics of the synthesized materials; while the two samples *x* = 0.4 and *x* = 0.5 represent a paramagnetic behavior. By relocating Ag^+^ to the B sites and removing Cu^2+^ from the A sites, the A–B superexchange interactions were suppressed, and the saturation magnetization diminished with increasing the Ag^+^ content.

The catalytic activity of the synthesized Cd_0.5_Cu_0.5−*x*_Ag_*x*_Fe_2_O_4_ nanoferrites in the model reaction of IC degradation by using H2O2 as an eco-friendly oxidant was evaluated. The catalytic reaction rate was fitted well by the first-order kinetics. The most active sample achieved 90% catalytic degradation of IC within 90 min. The catalytic activity was dependent on the Ag^+^ content and specific surface area. Moreover, the results reveal the benefits broke through the limitation of traditional Fenton reaction by pH range and significantly extended the application conditions of heterogeneous Fenton-based alkaline wastewater treatment. The catalytic process involved two basic redox couples Ag^+^/Ag^0^ and Cu^2+^/Cu^+^. Cd_0.5_Ag_0.5_Fe_2_O_4_ has a high catalytic ability, which can be ascribed to the presence of Ag^+^/Ag^0^ rather than Cu^2+^ since Ag^+^ has a higher electronegativity. These results provide a basic approach to better engineer and produce ferrite nanoparticles with expectable catalytic impact on AOP applications, nevertheless of whether these applications are in the field of industry or the environmental sector.

## Data Availability

All data generated or analyzed during this study are included in this article.
